# A Novel *TBX19* Gene Mutation in a Case of Congenital Isolated Adrenocorticotropic Hormone Deficiency Presenting with Recurrent Respiratory Tract Infections

**DOI:** 10.3389/fendo.2017.00064

**Published:** 2017-04-18

**Authors:** Nese Akcan, Nedime Serakıncı, Burcu Turkgenc, Ruveyde Bundak, Nerin Bahceciler, Sehime G. Temel

**Affiliations:** ^1^Faculty of Medicine, Department of Pediatric Endocrinology, University of Near East, Nicosia, Cyprus; ^2^Faculty of Medicine, Department of Medical Genetics, University of Near East, Nicosia, Cyprus; ^3^Genetic Diagnostic Center, University of Acıbadem, Istanbul, Turkey; ^4^Faculty of Medicine, Department of Pediatric Endocrinology, University of Kyrenia, Kyrenia, Cyprus; ^5^Faculty of Medicine, Department of Pediatric Allergy and Immunology, University of Near East, Nicosia, Cyprus; ^6^Faculty of Medicine, Department of Histology and Embryology, University of Near East, Nicosia, Cyprus; ^7^Faculty of Medicine, Department of Histology and Embryology, University of Uludag, Bursa, Turkey

**Keywords:** adrenal insufficiency, adrenocorticotropic hormone, cortisol, respiratory infections, *TBX19* gene

## Abstract

**Introduction:**

Congenital isolated adrenocorticotropic hormone deficiency (CIAD) is a rare disease characterized by low adrenocorticotropic hormone (ACTH) and cortisol levels. To date, recurrent pulmonary infections in infancy have not been reported as an accompanying symptom of CIAD.

**Case presentation:**

A 7-year-old boy was hospitalized nine times for recurrent lower respiratory tract infections. The results of all tests for the possible causes of wheezing were within the normal limits. His ACTH and cortisol levels were persistently low. All other pituitary hormone levels, and adrenal ultrasound and pituitary magnetic resonance imaging results, were normal. Molecular analyses confirmed the diagnosis of CIAD by identifying compound heterozygosity for two mutations in the *TBX19* gene. The first was a novel frameshift c.665delG variant in exon 4 of the *TBX19* gene, leading to premature termination that was predicted to result in a non-functional truncated protein. The second was a nonsense C-to-T transition in exon 6 of the *TBX19* gene, resulting in an arg286-to-ter mutation (dbSNP: rs74315376). Both parents were heterozygous for one of the mutations.

**Conclusion:**

Here, we presented a new mutation in the *TBX19* gene in a patient with CIAD who presented with recurrent respiratory tract infections. This expands the mutation spectrum in this disorder. To conclude, adrenal insufficiency should be considered in patients with unexplained recurrent infections to prevent a delay in diagnosis.

## Introduction

Congenital isolated adrenocorticotropic hormone deficiency (CIAD) is a rare disease characterized by low plasma adrenocorticotropic hormone (ACTH) and cortisol levels while the other pituitary hormone levels remain normal. CIAD occurs as a result of homozygous or compound heterozygous mutations in the *T-box 19* (*TBX19*) gene, which is located on chromosome 1q24 (1, 2).

Twenty-one different mutations were identified in the *TBX19* gene in the largest series (*n* = 91) of CIAD patients. Most CIAD cases present with severe hypoglycemia, seizures, or prolonged jaundice in the neonatal period (1). *TBX19* shares 94% amino acid identity with the mouse *Tpit* gene (2). Here, we report a CIAD case with a novel mutation presented with recurrent pulmonary infections. To our knowledge, this is the first reported case of CIAD presenting with recurrent pulmonary infection in infancy.

## Case Presentation

A 34-month-old boy was referred to our hospital with a pre-diagnosis of acquired ACTH deficiency. The patient was a term baby, delivered by cesarean section. He was intubated as a result of respiratory distress and supported by mechanical ventilation for 21 days in the neonatal intensive care unit. He was hospitalized nine times because of recurrent lower respiratory tract infections until the age of 10 months. In each period of hospitalization, he needed systemic steroid, and he also had continuously inhaled steroid during this period. Acquired ACTH deficiency secondary to glucocorticoid therapy or his medical conditions was decreed when the cortisol and ACTH levels were low at the age of 10 months. Hydrocortisone treatment (9 mg/m^2^/day) was started to prevent life-threatening complications. On admission to our hospital at the age of 34 months, he was on maintenance physiologic dose of hydrocortisone, his weight was 17.2 kg (+1.3 SDS), and his height was 97.5 cm (+0.5 SDS). He was normotensive and had natural skin and hair color, with normal male genitalia. Radiographic and biochemical analyses were normal. The cortisol response to a low-dose (1 µg) ACTH test was impaired (Table 1). The hydrocortisone treatment was continued with the same physiological replacement dose. Other causes of recurrent lung infection were ruled out by the Pediatric Allergy and Immunology Division. During follow-up, ACTH and cortisol levels were persistently low (ACTH < 5 pg/mL, cortisol < 1 ng/mL), and he had never demonstrated hypoglycemia or electrolyte imbalance. Growth hormone tests were not performed because of the normal stature. Levels of the other pituitary hormones (thyroid hormones, prolactin), 17-hydroxyprogesterone, androstenedione, and adrenal ultrasound and pituitary magnetic resonance imaging results were all normal (Table 1). Based on the hormonal profile, CIAD was suspected. On final assessment at 7 years 2 months, the patient weighed 28.3 kg (+1.1 SDS), and his height was 123.7 cm (+0.3 SDS). He has experienced no further lung infections since the hydrocortisone treatment was started.

**Table 1:** **Clinical findings and laboratory results**.

	On admission	Final examination
Age	34 months	7.2 years
Weight (kg)/weight-SDS	17.2/1.3	28.3/1.1
Height (cm)/height-SDS	97.5/0.5	123.7/0.3
BMI (kg/m^2^)/BMI-SDS	18/1.30	18.5/1.4
Tanner stage (pubic hair development)	1	1
Testes volumes	Testes 2/2 mL	Testes 2/2 mL
Laboratory tests (reference ranges)		
FBG (60–100 mg/dL)	65	90
Na (135–145 mmol/L)	142	140
K (3.5–5 mmol/L)	4.6	4.9
ACTH (6–48 pg/mL)	**<5**	–
Cortisol (3–21 μg/dL)	**0.9**	–
TSH (0.6–5.5 μIU/mL)	1.2	2.6
fT4 (0.85–1.75 ng/dL)	1.2	1
PRL (3–18 ng/mL)	16.1	16.3
DHEAS (13–83 μg/dL)	21	
A4 (10–17 ng/dL)	13	–
17α-OHP (<91 ng/dL)	11	–
Peak cortisol response to low-dose (1 µg) ACTH stimulation test (≥18 μg/dL)	**0.1**	–
Karyotype	46, XY	
Bone age (years)	2	7
Adrenal USG	Normal	
Cranial and pituitary MRI	Normal	

*ACTH, adrenocorticotropic hormone; A4, androstenedione; BMI, body mass index; DHEAS, dehydroepiandrosterone sulfate; FBG, fasting blood glucose; fT4, free thyroxine; K, potassium; Na, sodium; MRI, magnetic resonance imaging; PRL, prolactin; SDS, standard deviation score; TSH, thyroid-stimulating hormone; USG, ultrasonography; 17α-OHP, 17α-hydroxyprogesterone (abnormal findings are shown in bold)*.

In addition, the triple screening test of the mother during her second pregnancy showed low estriol (E3) and human chorionic gonadotropin (hCG) levels, which may be related to glucocorticoid deficiency. Genetic counseling was given to the pregnant woman. It was explained that early disease diagnosis allows for immediate commencement of glucocorticoid therapy, and proper instructions for stress management were provided. Although this can prevent unnecessary neonatal deaths from an easily treatable disease, in this case the family decided to terminate the pregnancy.

## Molecular Analyses

**Figure 1:**
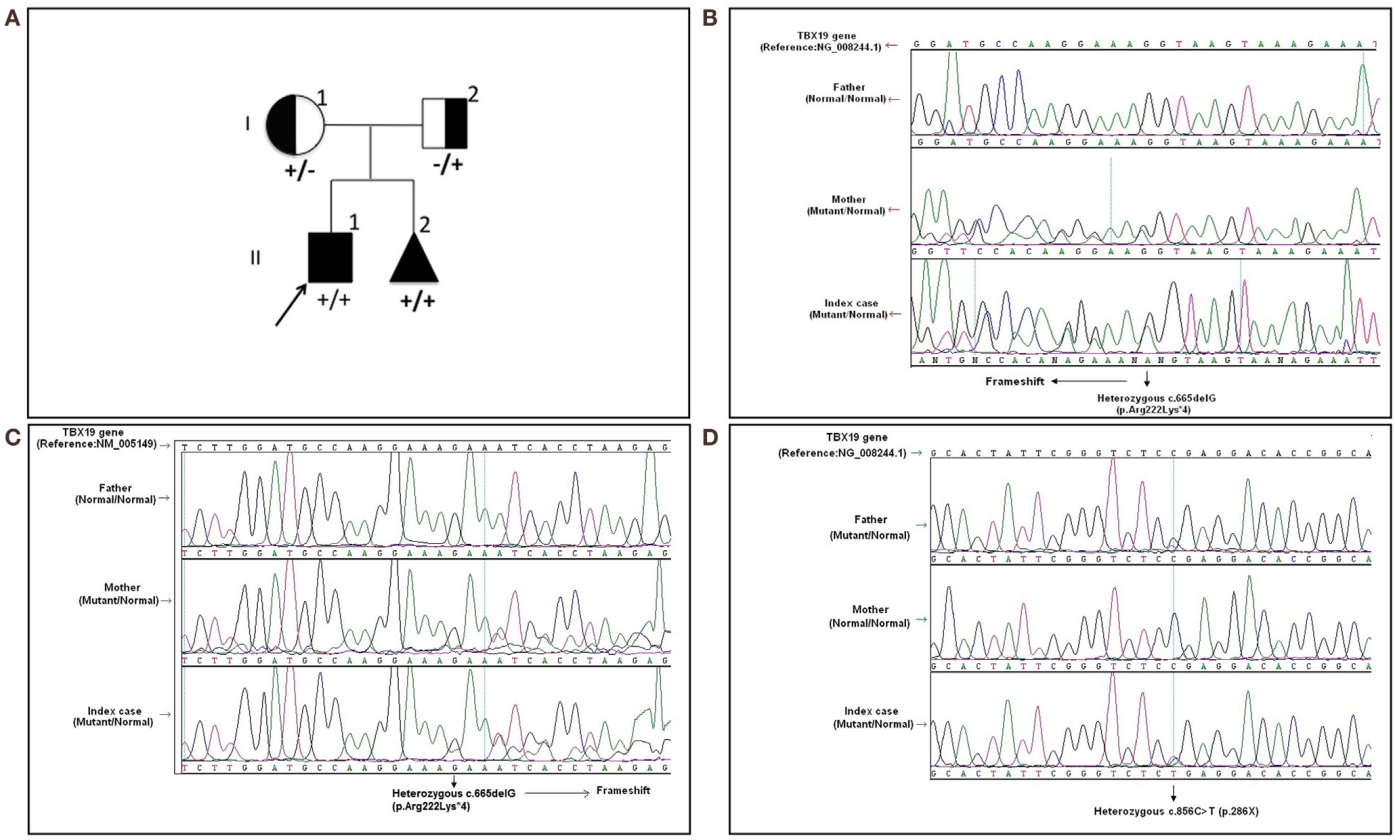
**The pedigree and mutations of “*TBX19* gene” detected in family members**. **(A)** The pedigree of family carrying *TBX19* mutations. **(B)** The image of heterozygous c.665delG (p.Arg222Lysfs*4) deletion in exon 4, which was inherited from the mother. The study was performed from the genomic DNA material. **(C)** The image of the same frameshift p.Arg222Lysfs*4 deletion studied from the RNA material of the index case and his carrier mother. **(D)** The image of known heterozygous c.856C>T mutation inherited from the father.

Written informed consent was obtained from the patient’s legal guardians. Molecular analysis was performed when he was 6 years old during the mother’s pregnancy with her second baby. Further molecular genetic testing of the parents was performed to assess whether both variants were on the same (*cis*) or a different (*trans*) *TBX19* allele and the abortion material was also analyzed molecularly. The pedigree was shown in Figure 1A.

In the molecular analysis of the index case, a novel heterozygous c.665delG (p.Arg222Lys*4) variation in exon 4 (Figures 1B,C) and pathologic heterozygous c.856C>T (p.Arg286Ter) variation in exon 6 (Figure 1D) of the *TBX19* gene (NM_005149.2, NP_005140.1) were detected in the compound heterozygous state. The initial genetic test suggested a possible splice site variation (c.665 + 1delG) in intron 4 of the *TBX19* gene instead of a c.665delG variant, because of the consecutive GG repeat at the exon–intron boundary. Therefore, further molecular analyses were performed at the mRNA level to gather information about the location of the variation (the last nucleotide of exon 4 or the first nucleotide of intron 4) and to identify a possible splicing effect. The mRNA analyses revealed that the real variation was c.665delG (p.Arg222Lysfs*4) and that it was a novel exonic frameshift mutation in spite of the splice site mutation (Figure 1C). As a consequence of the deletion of guanine 665 at the cDNA level, the first affected amino acid arginine was replaced with lysine at residue 222, and the new reading frame ended with a stop codon at position 4.

The other c.856C>T substitution was a nonsense variant predicted to result in the substitution of an arginine by a premature stop codon at position 286 in the protein (p.Arg286Ter) (Figure 1D). The c.856C>T variant was previously described in the Human Genome Mutation Database; it is associated with isolated deficiency of the pituitary and ACTH (HGMD #CM014746) (2). Also, it is listed as a pathogenic allele in the dbSNP (rs74315376) and ClinVar databases,^1^ with a lower minor allele frequency of 0.00005 (6 of 121,334 alleles).^2^

Molecular analysis revealed that the mother was a carrier of the c.665delG variation (Figures 1B,C) and the father was a carrier of the p.R286X mutation (Figure 1D). The abortion material also showed the same pathological variations in the *TBX19* gene.

## Discussion

Recurrent wheezing or pulmonary infections in infancy can be related to asthma, immune deficiency, congenital anatomic causes, and cystic fibrosis (3). However, recurrent pulmonary infections in infancy have not been reported as an accompanying symptom of any kind of GC deficiency. Although there is no relevant information regarding the relationship between cortisol insufficiency and recurrent infections, GCs play a critical role in restraining, shaping, and maintaining the homeostasis of the immune response (4, 5). Moreover, many reviews have focused on the bidirectional communication between the immune system and the HPA axis (4, 5). The ability of steroids to inhibit pro-inflammatory cytokines by switching off inflammatory genes through interactions with transcription factors but to enhance the anti-inflammatory cytokine may be the major effects of GCs, which may contribute to reduce the bronchial hyperresponsiveness (4–6).

The *TBX19* gene, previously known as *TPIT*, is a member of a phylogenetically conserved family of genes, which share a common DNA-binding domain: the T-box. *T-box* genes encode the transcription factors involved in regulating embryonic development. *TBX19* is a transcriptional regulator that interacts with target genes through its T-box domain (2, 7). *TBX19*-knockout mice had an almost complete lack of proopiomelanocortin-expressing cells, which resulted in severe ACTH and glucocorticoid deficiencies (8). Most of the mutations reported to date are clustered in the *T-box* region in the DNA-binding region of the gene, and as such might lead to loss of function. The T-box domain stretches between amino acids 45 and 218 and is essential for DNA binding.^3^ However, many of the mutant transcripts, such as R179X, R286X, and 782delA, which would be predicted to result in the premature truncation of the protein are reportedly destroyed by nonsense-mediated mRNA decay (9).

The *R286X* mutant transcript was one of the variations found in our case, in a heterozygous manner outside the *T-box* region. The mRNA transcripts resulting from the 573del4 mutant and the 5.2 kb deletion may be similarly destroyed or lead to severely truncated proteins (10). In contrast, the T58A, S128F, and I171T mutant transcripts produce defective proteins because these variations are within the T-box region. Therefore, the S128F and I171T mutant proteins exhibit no transcriptional activity or DNA-binding capacity, and the T58A mutant protein had greatly reduced levels of both transcription and DNA binding (9). This variant may lead to a truncated protein or reduce mRNA levels due to mRNA decay.

**Figure 2:**
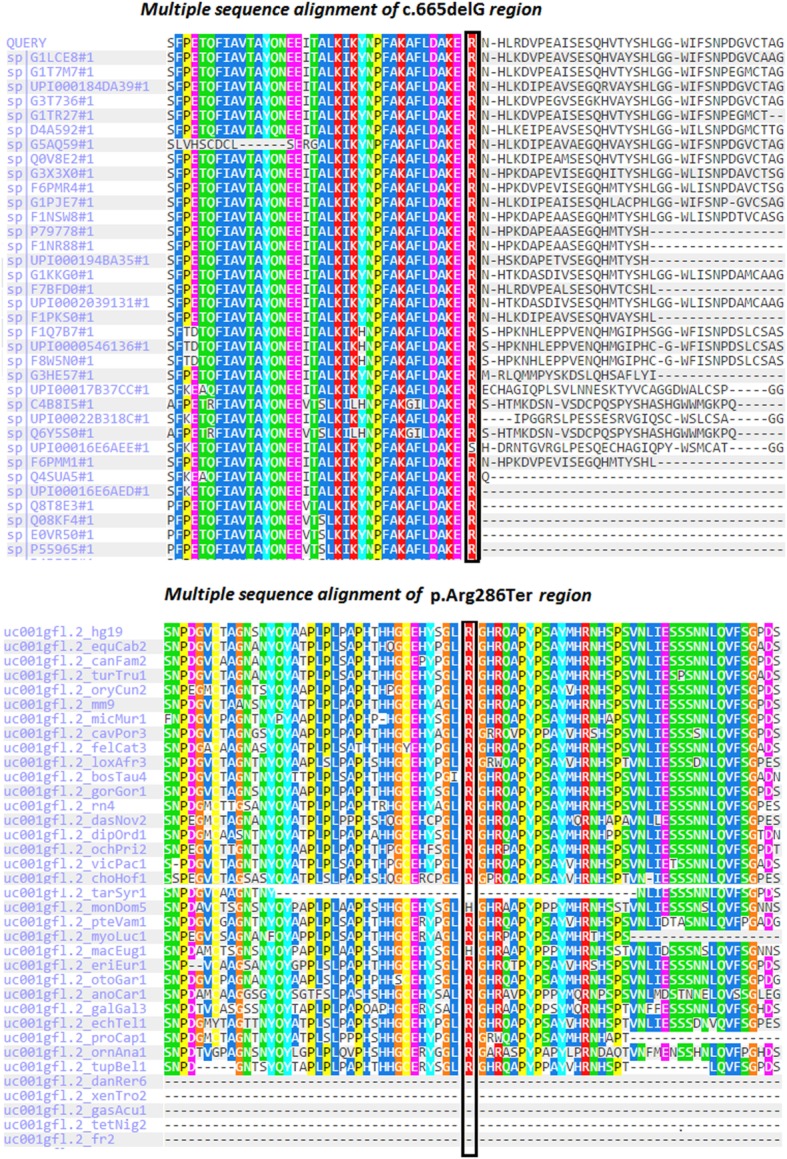
**Multiple sequence alignments of *TBX19* variants across different species due to PolyPhen-2 data**.

**Figure 3:**
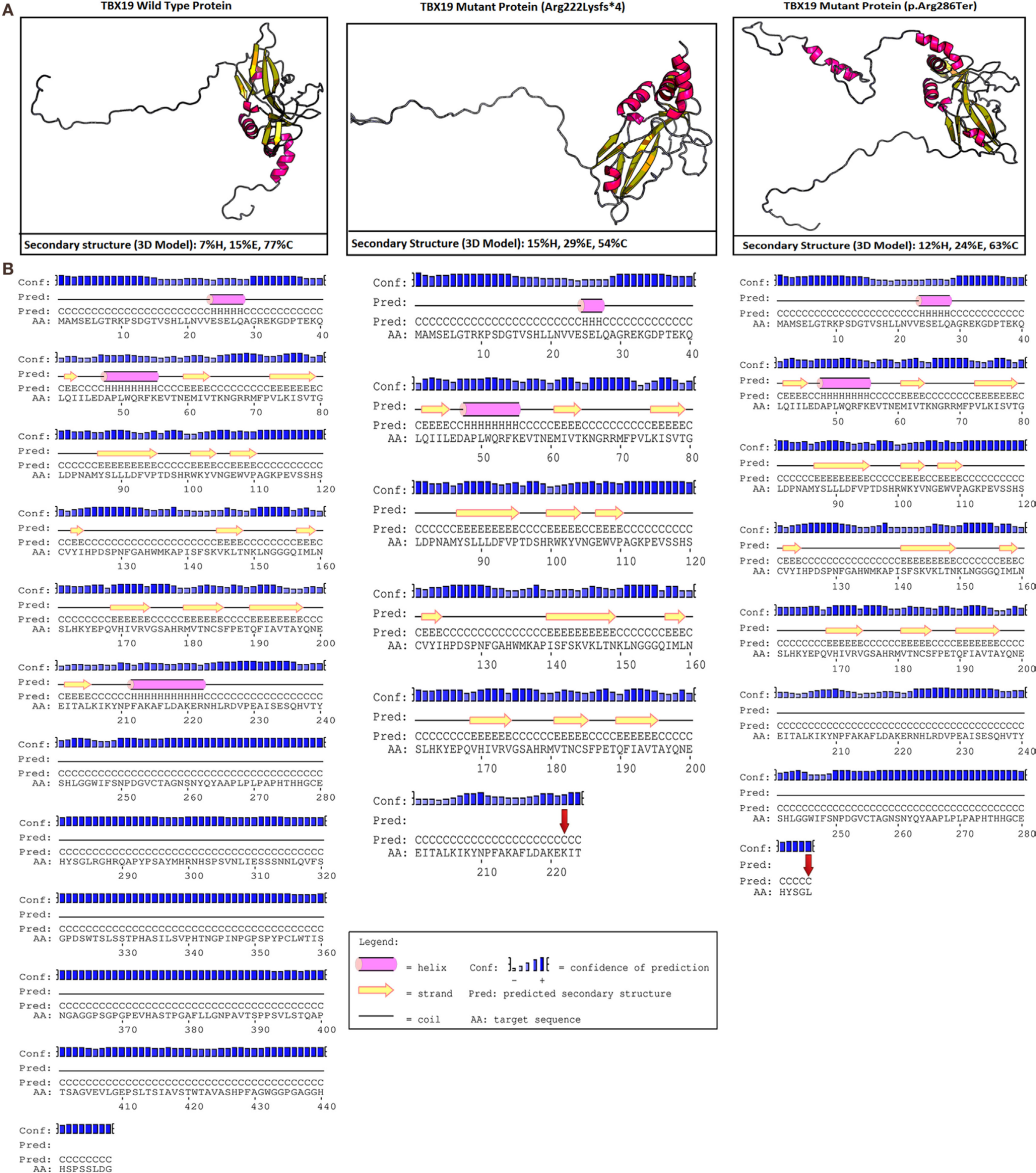
***In silico* analyses of wild type and mutant *TBX19* proteins**. **(A)** Predicted secondary structures and 3D modeling of wild type and mutant *TBX19* proteins (H, helix; E, beta sheet; C, loop). For 3-state secondary structure, it is predicted that the secondary structures of *TBX19* mutant types are altered when compared to wild type. **(B)** Predicted secondary structures of wild type and mutant *TBX19* proteins. The altered residues of c.665delG (p.Arg222Lysfs*4) and c.856C>T (p.Arg286Ter) are shown with red arrows. It is predicted that the secondary structures of *TBX19* mutant types are altered when compared to wild type.

In our case, c.665delG (Arg222Lysfs*4) was a novel heterozygous mutation that may not disrupt DNA binding, since this residue is outside the T-box region. Mutations in splice regions may lead to the retention of large segments of intronic DNA by the mRNA, or to entire exons being spliced from the mRNA. This led to a premature stop codon, shortening the protein. Conservation of the involved residues was examined across different species using the UCSC Genome Browser^4^ and PolyPhen-2.^5^ Multiple sequence alignments of different species showed that arginine-222 is highly conserved, whereas arginine-286 is relatively conserved (Figure 2). RaptorX^6^ and PSIPRED^7^ were used for *in silico* analysis of the effects of *TBX19* variants on the secondary structures of proteins. Comparison of the secondary and three-dimensional structures of the proteins revealed that helix, beta sheet, and loop structures were altered in both of the mutant proteins, which led to premature termination compared with the wild-type protein (Figures 3A,B).

In a series of 91 CIAD patients (the largest neonatal CIAD case series reported to date), among the 69 patients with neonatal-onset CIAD (37 and 32 patients with and without the *TPIT* mutation, respectively), 57 had complete ACTH deficiency and 12 had partial ACTH deficiency. In neonatal-onset cases with *TPIT* mutations, neonatal hypoglycemia was reported in all patients (37/37, 100%) whereas the frequency of other clinical signs, such as prolonged cholestatic jaundice and seizures, was 62% (21/34) and 53% (16/30), respectively. No recurrent respiratory infections were reported (1). However, the current case presented with recurrent respiratory infections. Interestingly, he had no hypoglycemia, seizures, or prolonged neonatal jaundice. It may be speculated that intermittent use of systemic steroids during infection may also prevent hypoglycemia and electrolyte imbalance, which could delay diagnosis in the early months. In addition, the novel and previously reported compound heterozygous mutations were both located outside of the DNA-binding domain and may be associated with the phenotype of the current case.

During the second pregnancy of the mother, low levels of E3 and hCG were detected. Low E3 levels in the context of normal fetal sonography and growth are reported in placental sulfatase deficiency and Smith–Lemli–Opitz syndrome where fetal steroidogenesis is defective. Low maternal E3 levels during pregnancy should raise suspicion of deficient fetal steroidogenesis (11, 12). Therefore, prompt evaluation of the infant for glucocorticoid deficiency in the first postnatal days will allow for early diagnosis.

In conclusion, adrenal insufficiency should be taken into account to prevent fatality and/or complications in infants with recurrent lung infections. Genetic analysis confirmed the diagnosis, and a new mutation was identified in our patient. We postulated that this novel and previously reported compound heterozygous mutations may be associated with recurrent lung infections in infants with CIAD. In addition, knowledge of the molecular mechanisms underlying this clinical entity could allow prenatal or early neonatal diagnosis in families at risk, and significantly prevent lethality.

## Patient Confidentiality

The patient’s guardian provided informed consent for publication of the submitted article, and the results of the accompanying genetic analyses, after a full explanation of the purpose and nature of all the procedures used.

## Author Contributions

NA: following-up the patient, clinical diagnosis, collecting all data, summarizing and writing main draft of the article. NS, BT, and ST: genetic analyses; writing and checking all the data in the article. RB: following-up the patient, clinical diagnosis, and writing and checking all the data in the article. NB: differential diagnosis for recurrent infections; writing and checking all the data in the article.

## Conflict of Interest Statement

The authors declare that the research was conducted in the absence of any commercial or financial relationships that could be construed as a potential conflict of interest.

## Footnotes

^1^https://www.ncbi.nlm.nih.gov/clinvar/variation/5440/.

^2^http://exac.broadinstitute.org/gene/ENSG00000143178.

^3^http://www.uniprot.org/uniprot/O60806.

^4^https://genome.ucsc.edu/cgi-bin/hgBlat.

^5^http://genetics.bwh.harvard.edu/pph2/.

^6^http://raptorx.uchicago.edu/StructurePrediction.

^7^http://bioinf.cs.ucl.ac.uk/psipred/.
